# In Vitro Comparison to Evaluate Metal Ion Release: Nickel-Titanium vs. Titanium-Molybdenum Orthodontic Archwires

**DOI:** 10.7759/cureus.56595

**Published:** 2024-03-20

**Authors:** Rana Chikhale, Pankaj Akhare, Utkarsh Umre, Rashmi Jawlekar, Shantanu Kalokhe, Neha Badole, Arushi Beri

**Affiliations:** 1 Orthodontics and Dentofacial Orthopaedics, Swargiya Dadasaheb Kalmegh Smruti Dental College, Nagpur, IND; 2 Endodontics, Sharad Pawar Dental College and Hospital, Datta Meghe Institute of Higher Education and Research, Wardha, IND; 3 Prosthodontics, Sharad Pawar Dental College and Hospital, Datta Meghe Institute of Higher Education and Research, Wardha, IND

**Keywords:** archwire, orthodontic, leaching of metal ions, niti, tma

## Abstract

Background

When metals used in orthodontic materials are exposed to the oral environment, teeth, and gingivae over an extended period of time, they may gradually deteriorate. As a result, the patient is exposed to higher concentrations of metals and metal ions than what they would be exposed to through food and other sources. The goal of the current in vitro experiment was to evaluate and contrast the metal ion release from orthodontic archwires made of titanium-molybdenum alloy (TMA) and nickel-titanium (NiTi).

Methods

For 90 days, 20 orthodontic archwires in each group were immersed in 50 milliliters of simulated saliva using different containers. Atomic Absorption Spectrometry (AAS) (Shimadzu Corporation, Kyoto, Japan) was used to assess and compare metal ion emission. The unit of measurement is parts per million (PPM).

Results

The findings indicated that the discharge of nickel metal from the NiTi archwire (Group A) was much higher than that from the TMA archwire (Group B), with a statistical significance level of p < 0.001. It was discovered that Group B's release of titanium was statistically significantly (p < 0.05) higher than Group A's titanium release, which did not include the release of any other metals.

Conclusion

The study findings indicated that the amounts of metal ions released from the orthodontic archwires made of titanium molybdenum and nickel-titanium alloy were within safety limits.

## Introduction

When nickel-titanium (NiTi) alloys were first introduced in 1971, orthodontic therapy made significant advancements [1]. In 1962, the Naval Ordinance Laboratory located in Silver Springs, Maryland, USA, was the birthplace of the NiTi alloys.

Gravina MA et al. were attracted to the distinctive features of these alloys, which include low modulus of elasticity, robust resilience, high elastic limit, and stiffness. These properties make the alloys well-suited for various applications, offering significant deformability without a substantial increase in stress, enduring repeated deformations without permanent damage, withstanding high-stress levels without experiencing permanent deformation, and maintaining resistance to deformation under applied loads, respectively. These characteristics have aided in the revival of novel techniques in orthodontics [2].

In general, 53.5% nickel, 44.9% titanium, and 1.6% cobalt make up these wire alloys [3]. Burstone invented the titanium-molybdenum alloy (TMA) in 1979, which revolutionised orthodontic therapy [1]. It combines good formability, a stiffness value closer to NiTi's than stainless steel's (roughly one-third as stiff), and acceptable resiliency. However, it suffers from surface roughness-induced friction and a higher propensity to fracture when creating loops. TMA wire has gained more traction than NiTi wire because of its superior qualities. TMA is made up of 4.3% tin, 6.6% zirconium, 11.3% molybdenum, and 77.8% titanium [4].

When metals used in orthodontic materials are exposed to the oral environment, teeth, and gingivae over an extended period, they may gradually deteriorate. As a result, the patient is exposed to higher concentrations of metals and metal ions than what they would be exposed to through food and other sources.

The estimated typical daily intakes of chromium and nickel from edible items are between 30 and 100 μg, and between 200 and 300 μg, respectively [3]. Titanium is biocompatible, but it is important to remember that nickel is known to be toxic so any exposure to it has to be closely managed. Stainless steel wires have around 8% nickel content, while NiTi alloys usually have a nickel concentration of more than 50% [1]. Due to its allergenic properties, nickel may cause hypersensitivity responses in certain people. Furthermore, carbonate salts, oxides, and sulfides of nickel have been classified as carcinogenic [5]. During the whole procedure, these alloys are inserted into the mouth cavity. Patients should be prepared to be exposed to some degree to the corrosion products of metal alloys due to the favorable conditions in the oral cavity that promote the biodegradation of metals such as ionic, thermal, microbiologic, and enzymatic properties. The oral environment contributes to the corrosion of metallic components in orthodontic equipment in several ways, including temperature (usually 37°C), protein content, bacterial flora, fluoride, pH of saliva, and enzyme activity [6].

When utilized in vivo, orthodontic components show indications of deterioration, such as morphological modifications and surface variations caused by wear, corrosion, and integument creation. When it comes to biocompatibility, nickel is distinct from other elements. The types of nickel used in dentistry have been related to carcinogenic, mutagenic, and cytotoxic qualities. However, no definite causal relationship has been shown between them and any local or systemic clinical disease [7].

If the quantities of excess metal seeping from orthodontic wires exceed the upper limits of daily consumption, hazardous responses may result. For example, it is recommended that daily consumption limits of no more than 300 µg of nickel, 0.2 mg of chromium, 5 mg of manganese, 3 mg of copper, 0.5 mg of molybdenum, 18 mg of iron, and trace amounts of cobalt be maintained. The normal daily consumption of titanium comes from food with an average of 300-2000 µg [8]. It is commonly known that the bulk of allergic responses in humans are caused by the metal nickel, with chromium following closely. It is important to remember that other metals included in orthodontic wires, such as molybdenum, titanium, manganese, cobalt, iron, and copper, can also harm the body if ingested in large amounts [9].

The number of research publications that look into the release of trace metals from orthodontic equipment has significantly increased recently. Numerous methods have been used to investigate these materials' biocompatibility and resistance to biodeterioration. Concerns about the probable toxicity of trace elements to patients and their prospective release during orthodontic treatment are the reason for this spike in study activities. Typically, orthodontic therapy uses a range of appliances composed of alloys that include cobalt, titanium, nickel, iron, and chromium, among other elements. However, nickel and chromium are the two metals that are concerning [10]. The objective of the current study was to compare and assess the levels of metal ion release from orthodontic archwires made of TMA and NiTi, taking into account the factors mentioned above. The intention was to highlight the actual consumption of caustic materials by orthodontic treatment patients.

## Materials and methods

Material

In this study, various materials were employed for experimentation. The first material utilized was artificial saliva, with a pH of 6.8, which was specially formulated within the biochemistry department of our institute. The composition of this artificial saliva consisted of sodium chloride (0.84 mg/100 mL), potassium chloride (1.2 mg/100 mL), magnesium chloride (0.052 mg/100 mL), calcium chloride (0.146 mg/100 mL), potassium dihydrogen phosphate (0.34 mg/100 mL), sorbitol solution (70%) at a volume of 60 mL, and hydroxyethyl cellulose (3.5 mg/100 mL).

The second material employed in the study was a 17 x 25-inch NiTi archwire, commonly used in modern orthodontics. This archwire was composed of 55% nickel and 45% titanium.

Additionally, a 17 x 25-inch TMA archwire (Modern Orthodontics, Ludhiana, India) was utilized. This wire was composed of 77.8% titanium, 11.3% molybdenum, 6.6% zirconium, and 4.3% tin. These materials were chosen for their relevance to orthodontic practice and their distinct compositions, allowing for comparative analysis within the study.

Study design and location

The objective of the current in vitro experiment was to measure and contrast the amount of metal ions emitted by orthodontic archwires made of TMA and NiTi alloys. The research was conducted at the Department of Orthodontics and Dentofacial Orthopaedics, Swargiya Dadasaheb Kalmegh Smruti (SDKS) Dental College and Hospital, Nagpur.

Study population

By using power analysis with G*Power, Version 3.0.1 (Franz Faul Universitat, Kiel, Germany), the sample size estimate was established. A total of 20 (10 units per wire type/group; two research groups) sample size units would provide 80% power to detect significant changes with an effect size of 1.2 at a 0.05 significance level. In vitro, purposeful sampling was used for 0.017 x 0.025-inch TMA and 0.017 x 0.025-inch NiTi orthodontic archwires (Modern Orthodontics). Inclusion criteria are new sterile orthodontic archwires. Exclusion criteria are used wires and broken wires.

Data collection

**Figure 1:**
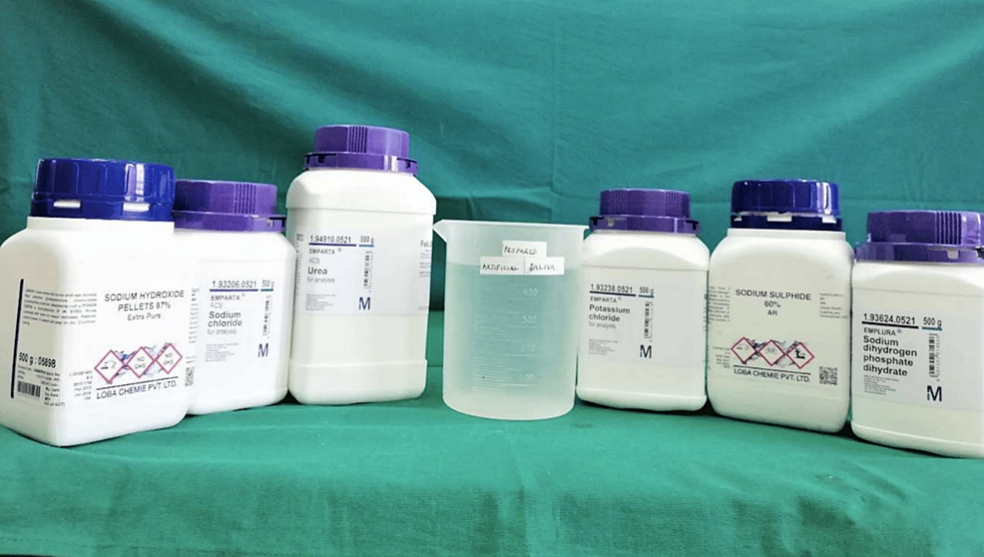
Artificial saliva prepared

**Figure 2:**
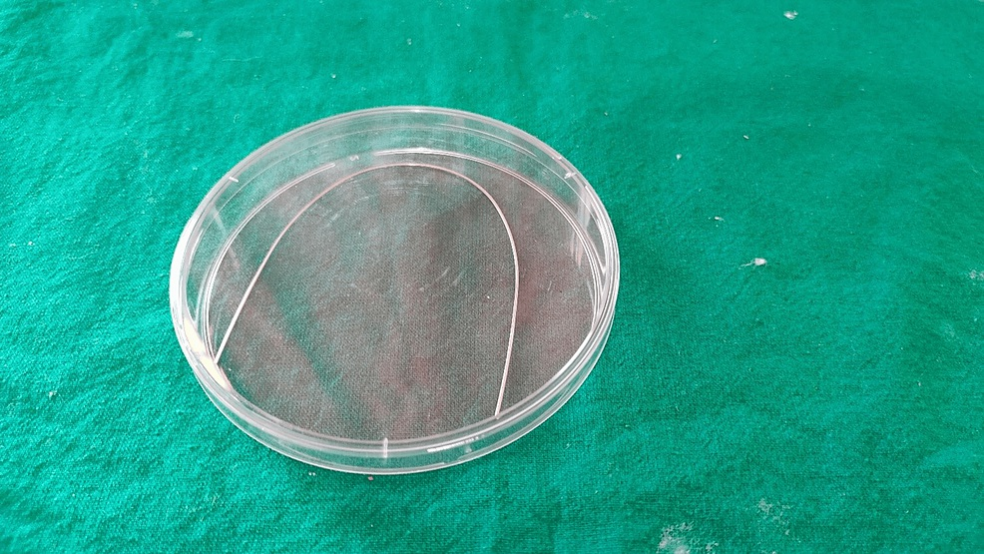
Archwire immersed in saliva

For this study, the orthodontic archwires were submerged in 50 mL of artificial saliva (amount: mg/100 mL): 0.052 mg magnesium chloride, 0.146 mg calcium chloride, 0.34 mg potassium dihydrogen phosphate, 60 mL sorbitol solution (70%), and 3.5 mg hydroxyethylcellulose, then placed in 10 distinct glass containers (Petri dishes) for 90 days for each group as shown in Figure 1 and Figure 2.

Using Atomic Absorption Spectrophotometry (AAS) (Model: Shimadzu AA-7000, optics: double beam with background correction, wavelength range: 190-900 nm), the artificial saliva in each glass container was collected after the 90-day incubation period. It was then subjected to analysis to quantify the release of metal ions in the saliva.

This study used AAS to examine the following metal ions: nickel, titanium, molybdenum, chromium, cobalt, tin, and zirconia. New sterile archwires were chosen, with old or damaged wires excluded. The unit of measurement of metal ions is parts per million (PPM).

Data analysis

The acquired data was statistically analyzed. SPSS Statistics for Windows, Version 21.0 (IBM Corp., Armonk, New York, United States) was used to conduct the statistical analysis. The mean and standard deviation were used to convey descriptive quantitative data. The Shapiro-Wilk test was used to determine if the data was normal. The likelihood of the alpha error (the level of significance) was set at 5%, and the confidence interval at 95%. The study's power was set at 80%. An unpaired t-test was used to compare the two types of archwires in terms of metal release between the groups.

## Results

**Figure 3:**
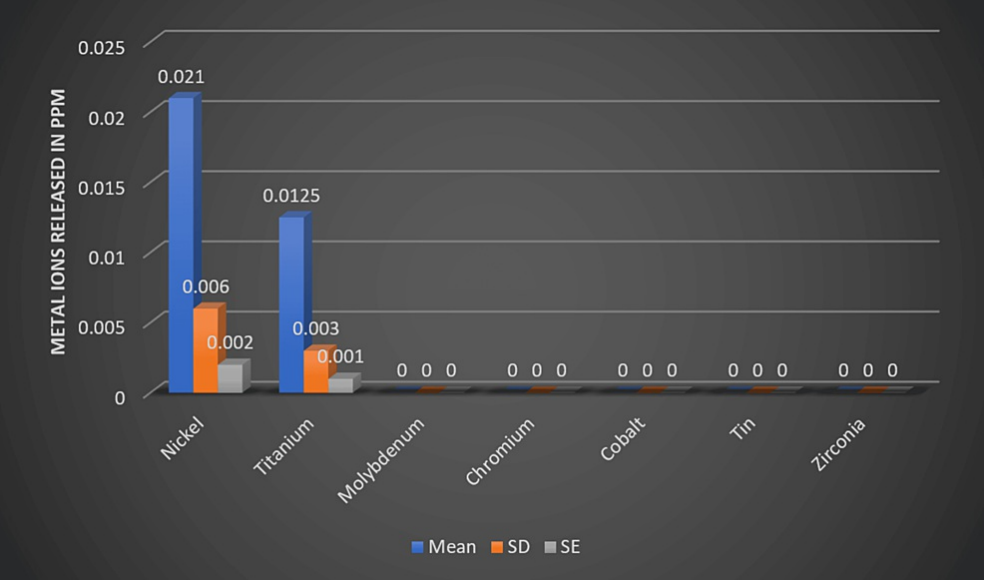
Metal release in PPM from NiTi archwire (Group A) PPM: Parts per million; NiTi: Nickel-titanium; SD: Standard deviation; SE: Standard error

The most amount of metals released was from the NiTi archwire. The highest release was from nickel, with a maximum of 0.021 PPM and a minimum of 0.002 PPM, with a mean deviation of 0.006 PPM. The second was from titanium, with a maximum of 0.0125 PPM and a minimum of 0.001 PPM, with a mean deviation of 0.003 PPM (Figure 3).

**Figure 4:**
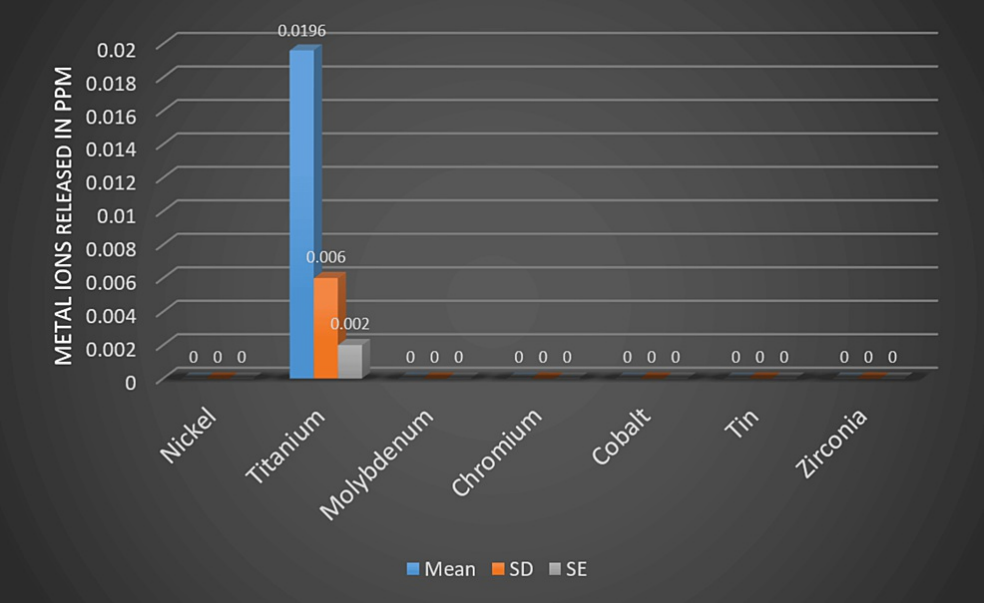
Metal release in PPM from TMA archwire (Group B) PPM: Parts per million; TMA: Titanium-molybdenum alloy; SD: Standard deviation; SE: Standard error

Release of titanium out of the rest with a release of 0.0196 to a minimum of 0.002 with a mean deviation of 0.006 (Figure 4).

Upon comparison of metals released from the NiTi archwire (Group A) and TMA archwire (Group B), nickel release was found to be highly statistically significantly (p < 0.001) higher in Group A as compared to Group B. Titanium release was found to be statistically significantly (p < 0.05) higher in Group B as compared to Group A. No release of molybdenum, chromium, cobalt, tin, or zirconia was observed in either Group A or Group B (Table 1).

**Table 1:** Comparison of metals released from the NiTi archwire (Group A) and the TMA archwire (Group B) Values mentioned in the table are measurements (SD) p > 0.05 – no statistical difference *p < 0.05 – significant **p < 0.001 – highly significant NiTi: Nickel-titanium; TMA: Titanium-molybdenum alloy; p-value: Probability value

	NiTi archwire (Group A)	TMA archwire (Group B)	Mean Difference	Unpaired t-test	p-value, Significance
Nickel	0.021 (0.006)	0.0 (0.0)	0.021 (0.002)	t = 9.996	p < 0.001**
Titanium	0.0125 (0.003)	0.0196 (0.006)	0.007 (0.002)	t = -3.157	p = 0.005*
Molybdenum	0.0 (0.0)	0.0 (0.0)	0.0	t = 0.0	p = 1.000
Chromium	0.0 (0.0)	0.0 (0.0)	0.0	t = 0.0	p = 1.000
Cobalt	0.0 (0.0)	0.0 (0.0)	0.0	t = 0.0	p = 1.000
Tin	0.0 (0.0)	0.0 (0.0)	0.0	t = 0.0	p = 1.000
Zirconia	0.0 (0.0)	0.0 (0.0)	0.0	t = 0.0	p = 1.000

## Discussion

It is crucial to ensure orthodontic equipment does not emit harmful ions when worn in the mouth for extended periods. The orthodontic appliances' components can deteriorate when exposed to various intraoral environments, such as saliva, and could result in the leakage of those components into the oral cavity. Inadvertent exposure to the discharged ions may result from this. Both in vitro and in vivo studies were conducted on the metal ion release from fixed orthodontic equipment, such as brackets and archwires [11,12].

When comparing the results with previous studies, it was found that the amount of metal ions leaked out from both the NiTi and TMA wires was similar. The concentration of leaked metal ions was within acceptable limits as defined by the Recommended Dietary Allowances (RDA), indicating that they are not harmful to humans unless there is a specific allergic reaction to a particular metal. This finding is particularly relevant in the context of concerns about nickel allergies.

Metals biodegrade when orthodontic appliances are in constant, long-term contact with teeth, gingivae, and the oral environment in general, especially saliva. When saliva is present, these appliances can become rougher, the friction between the archwires and the slot increases, and metal or alloy ions are released. It can cause localized discomfort, allergic responses in susceptible patients, and discoloration of the soft tissues and enamel. Due to their higher number of side effects, it has been demonstrated that nickel and chromium (Cr) are the components thought to be the most essential in corrosion products [13]. Orthodontic equipment may have detrimental biological effects based on the type and amount of metal ions it releases. In conventional orthodontic therapy, NiTi alloy orthodontic wires composed of about equal amounts of titanium and nickel are among the most often used types. This is due to their beneficial functional and mechanical characteristics [14].

By using artificial saliva in the present study, a controlled environment was created, which allowed for the assessment of ions released by orthodontic archwires. This information can help identify the potential risks and benefits of using different types of archwires in orthodontic treatment. Moreover, the comparison between NiTi and TMA archwires contributed to the existing literature and offers insights into the relative safety and efficacy of the materials. The results could influence the selection of archwires in orthodontic treatment planning and guide clinical decision-making. In general, the use of artificial saliva in this study provided a useful tool for investigating the orthodontic appliances' effects on the oral health environment.

The present study aimed to analyze the release of two common metal ions from NiTi and TMA archwires and compare them statistically. The results showed that the NiTi archwire (Group A) released nickel with a mean of 0.021 and titanium with a mean of 0.0125. On the other hand, the TMA archwire (Group B) released titanium with a mean of 0.0196. The remaining metals were either negligible or not present in both groups. According to the statistical study, Group A's nickel leak was noticeably greater than Group B's. In the same way, Group B's titanium leak was noticeably greater than Group A's. These results offer important information on the metal ion discharge from these archwires and how it can affect patients' dental health.

In 2001, Agaoglu et al. investigated the release of ions from fixed orthodontic equipment for one week, one month, one year, and two years using both blood and saliva. They discovered that saliva samples had the highest concentrations of metal ions during the first month following appliance insertion and that these levels had subsequently dropped to those observed before orthodontic treatment. In their investigation, saliva contained 4.12-11.53 ng/mL of nickel ion [15]. In 2004, Eliades, T. et al. studied the release of nickel and chromium, which was evaluated using stainless steel with NiTi. They found no measurable nickel and chromium traces detected for NiTi wires for a month. They analyzed the metal ions concentration by inductively coupled plasma atomic emission spectroscopy (ICP-AES), which was below the detection limit of that instrument. However, in the present study, it was found that the maximum amount of 0.03 units of nickel ion release in NiTi wires was due to a difference in the duration [16]. In 2006, Fors and Persson filtered saliva to assess the nickel release within 48 hours and found 0.005 μg/gram of nickel in the saliva [17].

In 1997, Kerosuo and Hensten-Pettersen conducted a study spanning one to two days, one week, and one month. Utilizing fixed equipment, they quantified the emission of nickel and chromium in saliva and concluded that there was no appreciable increase in nickel and chromium ions [18]. In 2008, Matos de Souza and Macedo de Menezes conducted a similar investigation. After collecting saliva over 60 days, they observed a significant increase in iron, nickel, and chromium levels. Specifically, a nickel ion release of 1.7-16 ng/mL was noted. Notably, their findings revealed an immediate rise in iron and nickel levels on the first day of the study [19]. In 2008, Petoumeno et al. evaluated nickel concentration in the saliva of orthodontic treatment undergoing subjects. Samples were taken before and after two, four, and eight weeks of placing NiTi archwires. They concluded the same nickel release level increased immediately after placement and then decreased, which was up to 28-78 ng/mL [20].

In 2008, Amini et al. used atomic absorption spectrometry to examine nickel, chromium, and cobalt levels in oral mucosal cells from individuals with and without permanent orthodontic apparatus. The study found that the average nickel content in the mucosal sample of orthodontic patients was 21.74 ng/mL [21]. In 2003, Faccioni et al. conducted a parallel investigation on oral mucosal cells using orthodontic equipment, yielding comparable results. Their findings revealed that the elevated concentration of nickel and cobalt released by the appliances led to DNA damage and a reduction in the proliferative capacity of mucosal cells. Notably, during the initial four to five months of the study, a total of 2.521 ng/mL of nickel ions was released [22].

In 2002, Huang et al. studied the release of nickel and titanium ions from NiTi orthodontic wire after it was submerged in artificial saliva for one to 28 days. Four kinds of NiTi wires were used in four distinct pH settings for the investigation. According to the data, the accumulation of nickel and titanium ions rose with longer immersion times. Additionally, the study discovered that compared to the first immersion test, the average daily release of nickel ions during the 28-day test was lower. On the 28th day, it was discovered that the release of nickel and titanium ions was 57.9 and 32.4 µg/cm^2^, respectively, at pH 2.5. The release of titanium ions was mostly undetectable at pH levels greater than 3.75 [14]. In 2009, Kuhta et al. carried out a study using two orthodontic devices that used thermos NiTi for 28 days at two different pH levels. According to the study, the first week of the appliances' immersion was when the most ions were discharged. The findings demonstrated that at pH 3.5, stainless steel wire produced the most copper ions, whereas NiTi wires released the least titanium ions at the same pH [23].

In 2019, Olszewska et al. conducted a study testing elastomeric and stainless steel ligatures in simulated human saliva over one, three, and six months. Among the 23 elements studied, cobalt, chromium, nickel, and tin were consistently released in both ligature types. Additionally, elastomeric ligatures showed the presence of cadmium and manganese, while stainless steel ligatures contained iron. In elastomeric ligatures, nickel was observed with a mean value of 0.25. In stainless steel ligatures, nickel release varied with mean values of 2.3, 2.9, and 5.0 observed at the one, three, and six-month marks, respectively. It indicated a progressive increase in nickel release throughout the study [12].

Nickel allergy, a prevalent condition impacting a substantial portion of the populace, is estimated to affect 10-15% of individuals based on research findings. Clinical manifestations of this allergy encompass skin-related symptoms such as irritation, redness, itching, rash, and, in severe instances, blistering. In the context of orthodontics, exposure to nickel-containing appliances can elicit these symptoms in the oral mucosa. One preventive measure involves the utilization of orthodontic archwires with alternative compositions devoid of nickel. However, achieving and maintaining the advantageous properties of NiTi archwires with alternative compositions pose challenges. Despite these difficulties, the TMA orthodontic archwire emerges as a promising alternative to NiTi archwires. Despite the known prevalence of nickel allergy, there is a notable absence of comprehensive studies detailing the specific quantity of nickel requisite for eliciting allergic reactions. Therefore, the establishment of conclusive guidelines regarding nickel threshold levels for allergic responses remains an area warranting further research. The current investigation demonstrates the mean of 0.0196 units of titanium ion release from the TMA archwires (Group B). Considering that there is not much research on the subject, the TMA archwires are a new development.

Since most research compared NiTi wire with stainless steel wires, TMA wires were used in the current study's comparison. When the metals released from the NiTi archwire (Group A) and TMA archwire (Group B) were compared, it was discovered that Group A's nickel release was significantly (p < 0.001) higher than Group B's nickel release. It was also discovered that Group B's titanium release was statistically significantly (p < 0.05) higher than Group A's titanium release. Additionally, it was discovered that neither Group A nor Group B had any releases of other metals, such as molybdenum, chromium, cobalt, tin, or zirconia. No other metals were released, according to the current investigation.

The current study's addition to understanding the metal ion release from NiTi and TMA archwires in the oral environment makes it clinically significant. According to the study, there was little metal ion leakage suggesting that both archwires may be used safely for an extended period during orthodontic therapy. Orthodontists can utilize this information to help choose the right type of archwire for patients. This study further emphasized how crucial it is to keep an eye on the metal ion discharge from orthodontic equipment to guarantee patient safety and well-being.

Limitation

The present study has certain limitations that need to be considered. Firstly, the use of artificial saliva may not entirely replicate the oral environment. The fixed pH of artificial saliva may not be representative of the fluctuating pH of the oral cavity. Secondly, the detection of ion release was conducted at fixed time intervals and not continuously, which may have missed any rapid changes in ion release. Lastly, the study did not investigate the release of ions after the complete treatment period due to time constraints. These limitations suggest that further studies with more comprehensive and accurate experimental designs are necessary to fully understand the behavior of orthodontic archwires in the oral environment.

## Conclusions

Based on the findings of this investigation, the metal ions released from both NiTi and TMA orthodontic archwires were within safety limits. The study suggests a variation in the quantity of nickel and titanium ions emitted by these two orthodontic archwire types. In summary, the study concludes that the amounts of metal ions released from orthodontic archwires made of NiTi and TMA were found to be safe. The primary concern remains with metal ion allergies in specific individuals, particularly nickel allergies, which are more common. However, aside from allergic concerns, it was deemed safe to use both types of wires. Therefore, there is no appreciable danger of exposure to hazardous amounts of metal ions when using any archwire type in the oral environment for an extended period.
